# A Feature Engineering Method for Whole-Genome DNA Sequence with Nucleotide Resolution

**DOI:** 10.3390/ijms26052281

**Published:** 2025-03-04

**Authors:** Ting Wang, Yunpeng Cui, Tan Sun, Huan Li, Chao Wang, Ying Hou, Mo Wang, Li Chen, Jinming Wu

**Affiliations:** 1 Agricultural Information Institute, Chinese Academy of Agricultural Sciences, Beijing 100081, China; wangting01@caas.cn (T.W.); lihuan@caas.cn (H.L.); houying@caas.cn (Y.H.); wangmo@caas.cn (M.W.); chenli02@caas.cn (L.C.); wujinming@caas.cn (J.W.); 2 Key Laboratory of Agricultural Big Data, Ministry of Agriculture and Rural Affairs, Beijing 100081, China; 3 Digital Agriculture and Rural Research Institute, Chinese Academy of Agricultural Sciences, Zibo 255035, China

**Keywords:** feature construction, genetic selection, Omics analysis, large language model, agronomic trait prediction

## Abstract

Feature engineering for whole-genome DNA sequences plays a critical role in predicting plant phenotypic traits. However, due to limitations in the models’ analytical capabilities and computational resources, the existing methods are predominantly confined to SNP-based approaches, which typically extract genetic variation sites for dimensionality reduction before feature extraction. These methods not only suffer from incomplete locus coverage and insufficient genetic information but also overlook the relationships between nucleotides, thereby restricting the accuracy of phenotypic trait prediction. Inspired by the parallels between gene sequences and natural language, the emergence of large language models (LLMs) offers novel approaches for addressing the challenge of constructing genome-wide feature representations with nucleotide granularity. This study proposes FE-WDNA, a whole-genome DNA sequence feature engineering method, using HyenaDNA to fine-tune it on whole-genome data from 1000 soybean samples. We thus provide deep insights into the contextual and long-range dependencies among nucleotide sites to derive comprehensive genome-wide feature vectors. We further evaluated the application of FE-WDNA in agronomic trait prediction, examining factors such as the context window length of the DNA input, feature vector dimensions, and trait prediction methods, achieving significant improvements compared to the existing SNP-based approaches. FE-WDNA provides a mode of high-quality DNA sequence feature engineering at nucleotide resolution, which can be transformed to other plants and directly applied to various computational breeding tasks.

## 1. Introduction

Genomic selection (GS) is an advanced method for enhancing breeding efficiency, integrating a wide array of genetic markers across the genome to forecast phenomics [1]. Approaches based on machine learning and deep learning have shown substantial improvements over traditional methods in predicting the phenotypic traits of plants. However, due to limitations in effectively processing large volumes of genetic data and understanding the relationship between different nucleotides in DNA sequences, these methods are primarily restricted to analyses based on single-nucleotide polymorphism (SNP) arrays [2,3,4].

While widely used in genomic studies, SNP arrays have inherent limitations that may affect the accuracy of their trait predictions. One critical limitation is that SNP arrays primarily rely on linkage disequilibrium (LD) to detect genetic signals, as they do not directly capture the truly causative variants underlying important phenotypic traits. Instead, SNP arrays sample a subset of variants that are, at best, in LD with the causative variants. This reliance on LD means that the resolution of association studies is constrained by the extent and structure of LD in the population, which can vary across genomic regions and between populations. Moreover, due to the design and cost constraints of genotyping technologies, many SNPs that are closely linked to trait-controlling genes may remain unmapped or poorly represented [5], further limiting the ability to identify causal loci. These limitations highlight the need for approaches that go beyond SNP array data, such as whole-genome sequencing, to directly capture all variants, including rare and non-coding variants that may play critical roles in trait determination [6,7]. SNP data also fail to consider the interactions between different nucleotide sites in DNA, as well as between nucleotides and amino acids, such as the interactions between DNA and histones [8]. These interactions profoundly influence protein expression, which in turn affects the accuracy of plant phenotypic trait predictions.

Analyzing whole-genome sequences at the nucleotide level allows for a comprehensive understanding of all of the genetic information contained within the genome, facilitating further improvements in the accuracy of genomic predictions [9]. However, the challenge lies in how to effectively analyze whole-genome DNA data.

The similarity between DNA sequences and natural language offers novel insights and methods for genetic sequence analysis [10,11,12]. DNA sequences, which have fixed rules and structures, are composed of basic symbols similar to those of language. There are also interactions and influences between nucleotides, which is consistent with the principles of natural language. If we apply the transformative methods in natural language processing (NLP) to the analysis of DNA sequences, such as transformer and large language models (LLMs), they could help us towards a deeper understanding of the intricate functions and structures of DNA sequences. Both academia and industry have already initiated related research [13,14,15,16]. The protein language model AlphaFold2, which is a typical example, achieved an unprecedented victory in the CASP14 protein structure prediction competition, demonstrating astonishing accuracy and efficiency. AlphaFold2 raised the accuracy from 42%, the best result in the previous competition, to 92%, marking an unparalleled advancement in the history of protein structure prediction [17].

The ongoing progress in LLMs, coupled with the rapid development of computational power, has also enabled trait prediction based on genome-wide DNA sequence analysis at nucleotide resolution, offering unprecedented opportunities to elucidate the relationship between genomic information and phenotypic traits. Researchers have proposed LLM-based models in genomics to learn generalizable features from unlabeled DNA sequences that can then be fine-tuned for downstream tasks, such as identifying regulatory elements. DNABERT is one of these genomic foundation models, but it relies on attention-based transformers, resulting in there being only 512 to 4096 tokens as context [18], which significantly limits the modeling of long-range interactions in DNA. In addition, these methods rely on tokenizers or fixed k-mers to aggregate meaningful DNA units, losing single-nucleotide resolution.

Recently, Hyena, an LLM based on implicit convolutions, was shown to match attention in quality while offering longer context lengths and lower time complexity. Leveraging Hyena’s new long-range capabilities, HyenaDNA achieved groundbreaking progress in classifying long DNA sequences by leveraging fine-tuned Hyena on human reference genome data, while using significantly fewer model parameters [19]. However, the HyenaDNA method primarily focuses on processing one human whole-genome sequence, and it outputs the feature construction for only a partial DNA sequence without providing a scheme for whole-genome DNA sequence. To address this limitation, we adapted and improved the HyenaDNA method to construct a feature engineering framework for plant whole-genome DNA sequences, using soybeans as an example.

In this study, we propose a novel whole-genome DNA sequence feature construction model, FE-WDNA, which leverages the LLM framework of HyenaDNA that was fine-tuned on plant genomes. First, we introduce FE-WDNA, followed by a performance comparison with several existing methods in genomic selection. We also analyze the factors that affect FE-WDNA, including the DNA sequence input style, the fusion mode of feature vectors from different chromosomes, and so on.

Our contributions are summarized as follows: (1) By analyzing the whole-genome DNA sequence at nucleotide resolution, FE-WDNA optimizes the feature construction process for genomic prediction in plant breeding. (2) To the best of our knowledge, this study represents the first application of LLMs to feature construction for plant whole-genome analysis and the first to utilize complete nucleotide sequences for trait prediction. (3) We implement FE-WDNA in a soybean phenotypic trait prediction task, achieving significant improvements compared to current state-of-the-art (SOTA) methods.

## 2. Results

We analyzed the performance of FE-WDNA in soybean trait prediction by comparing it with existing state-of-the-art (SOTA) methods in genomic prediction. First, we evaluated the performance of FE-WDNA in both qualitative trait prediction, using accuracy as the metric, and quantitative trait prediction, using MAE, MSE, and PCC as metrics. Next, we investigated various factors influencing the performance of FE-WDNA, including the input format and window length of DNA sequences, the feature vector dimensions and construction method for the whole-genome DNA sequence of a plant sample, and the sample size used for model training.

### 2.1. Performance Comparison Between Different Trait Prediction Methods

We compared the performance of quantitative and qualitative trait prediction based on FE-WDNA with that of SoyDNGP, DeepGS, and DNNGP. Their performance for seven quantitative traits including plant height (PH), oil content, protein content, flowering time (FT), maturity time (MT), yield, and hundred-seed weight (HSW) was assessed using MAE, MSE and PCC as the indicators. Performance for four qualitative traits—flower color (FC), stem termination (ST), pod color (POD), and pubescence density (PDENS)—was evaluated using accuracy as the indicator.

#### 2.1.1. Quantitative Trait Prediction

**Figure 1:**
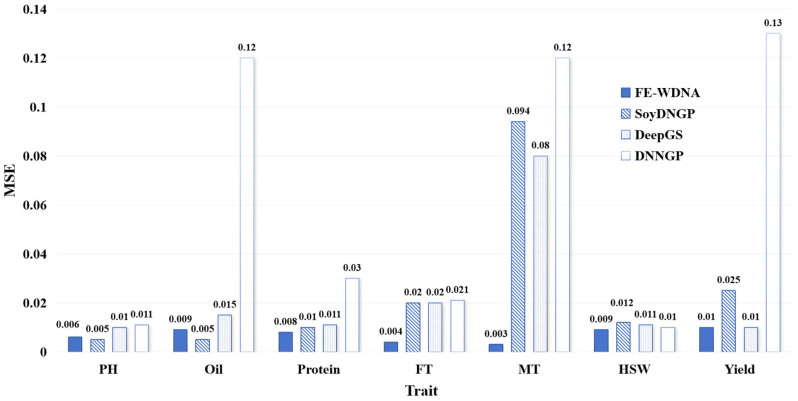
Comparison of quantitative trait prediction with FE-WDNA and the existing methods based on SNP data using the mean square error (MSE) as the metric. PH, plant height; FT, flowering time; MT, maturity; HSW, hundred-seed weight.

The MSE values for quantitative trait prediction using different methods are presented in Figure 1. Blue solid bars represent FE-WDNA, diagonal striped bars indicate SoyDNGP, dotted bars represent DeepGS, and hollow bars represent DNNGP. The same bar style is applied across all comparison figures for other traits. For the trait PH, SoyDNGP achieves the lowest MSE, but the difference between FE-WDNA and SoyDNGP is only 0.01. DeepGS and DNNGP exhibit significantly poorer performance. For the trait Oil, SoyDNGP performs the best, while FE-WDNA shows moderate performance. DNNGP performs the worst, with an MSE of 0.12, which is substantially higher than SoyDNGP’s MSE of 0.005. For the trait Protein, FE-WDNA outperforms all methods, followed by SoyDNGP and DeepGS. DNNGP shows the poorest performance. For the traits FT and MT, FE-WDNA achieves the best results with MSE values of 0.004 and 0.003, respectively, which are significantly lower than the minimum values of 0.02 and 0.08 achieved by the other three methods. For the trait HSW, the performance of the four methods is comparable, with FE-WDNA achieving a slightly lower MSE. For the trait Yield, FE-WDNA and DeepGS exhibit the best performance, with identical MSE values. Overall, FE-WDNA demonstrates stable performance across all traits compared to the other three methods, maintaining MSE values below 0.01 in most cases. The only exceptions are the traits PH and Oil, where FE-WDNA’s MSE is slightly higher than that of SoyDNGP.

**Figure 2:**
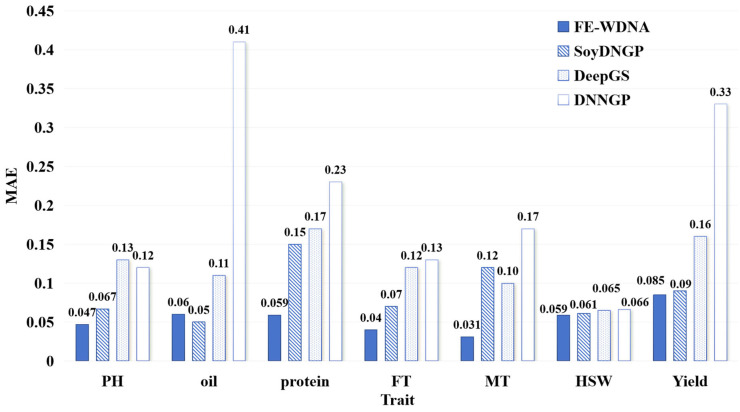
Comparison of quantitative trait prediction with FE-WDNA and the existing methods based on SNP data using the mean absolute error (MAE) as the metric. PH, plant height; FT, flowering time; MT, maturity time; HSW, hundred-seed weight.

The MAE values for the different prediction methods are shown in Figure 2. For the trait PH, the MAE of FE-WDNA is consistently lower than those of the other three methods, outperforming it based on the MSE metric. This may be due to FE-WDNA producing relatively uniform prediction errors, whereas SoyDNGP, DeepGS, and DNNGP exhibit extreme outliers with large errors. For the trait Oil, the MAE of FE-WDNA is slightly higher than that of SoyDNGP but remains significantly lower than those of the other two methods. For the remaining five traits, FE-WDNA achieves the lowest MAE values across the board, followed by SoyDNGP and DeepGS, which show comparable performance in most cases. DNNGP consistently performs the worst, with both its MAE values and the variation in MSE being notably higher than those of the other methods. Based on the combined evaluation of the MSE and MAE metrics, FE-WDNA excelled in most cases and exhibited a well-balanced overall performance, demonstrating strong generalization capabilities in predicting soybean quantitative traits.

**Figure 3:**
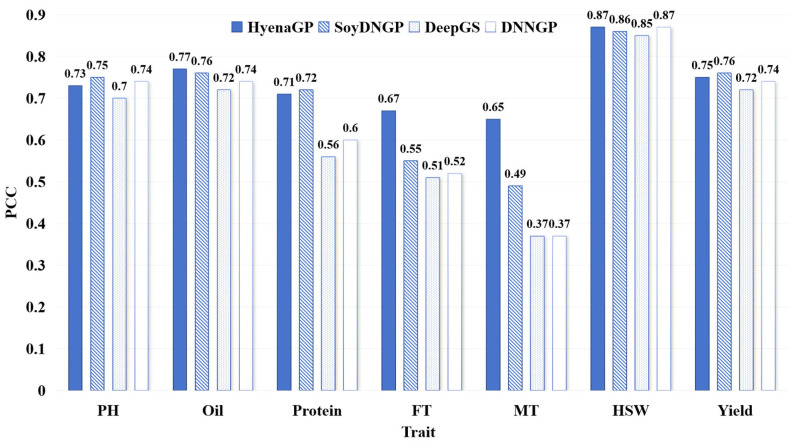
Comparison of quantitative trait prediction based on FE-WDNA and the existing methods based on SNP, using Pearson’s correlation coefficient (PCC) as the metric. PH, plant height; FT, flowering time; MT, maturity time; HSW, hundred-seed weight.

The PCC values for different phenotypic trait prediction methods are shown in Figure 3. For traits such as Oil, FT, MT, and HSW, FE-WDNA outperforms the other methods, particularly for FT and MT, where its PCC values exceed 65%, representing improvements of 12% to 28% over the other methods, respectively. For the other three traits, the PCC values of FE-WDNA are slightly lower than those of the best-performing SoyDNGP. Overall, the advantage of FE-WDNA for PCC is less pronounced than for MAE and MSE but still reflects its capability in quantitative trait prediction.

#### 2.1.2. Qualitative Trait Prediction

**Figure 4:**
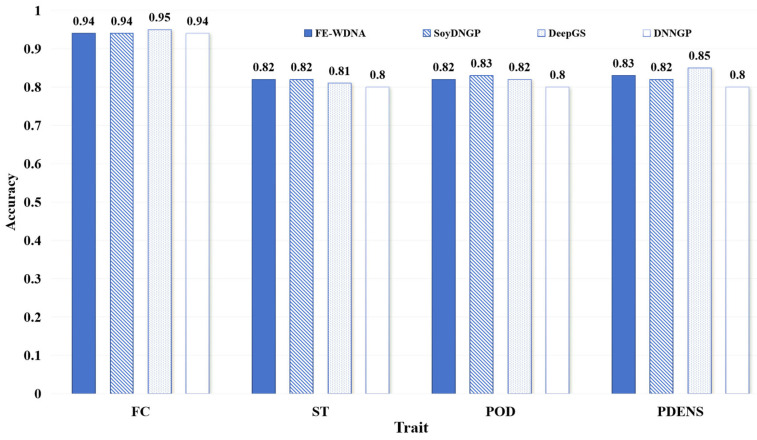
Comparison of qualitative trait prediction based on FE-WDNA and the existing methods based on SNP data. Soybean dataset. FC, flower color; ST, stem termination; POD, pod color; PDENS, pubescence density.

The performance comparison of different methods for qualitative trait prediction is shown in Figure 4. The differences among the four methods are not substantial, with FE-WDNA differing from the optimal values by only 1% to 2%. This indicates that FE-WDNA performs comparably to the existing methods in qualitative trait prediction tasks.

### 2.2. Exploration of Factors Influencing the Trait Prediction Performance with FE-WDNA

#### 2.2.1. Different DNA Sequence Input Modes of FE-WDNA

**Figure 5:**
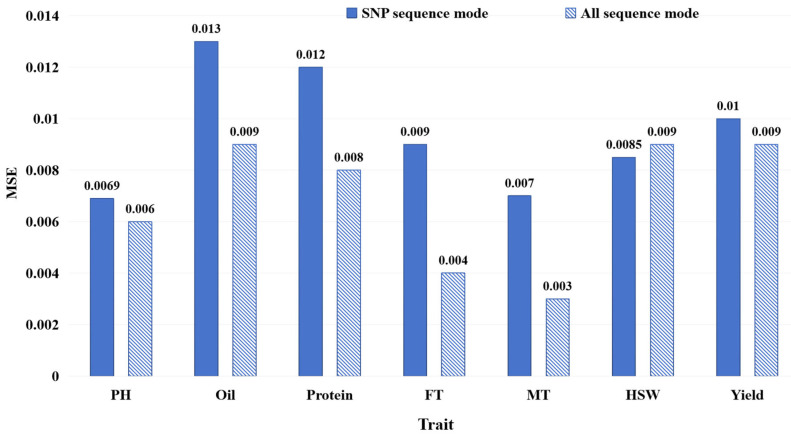
MSE of FE-WDNA with different DNA sequence input modes.

We conducted performance comparisons between the all-sequence mode and the SNP sequence mode, as illustrated in Section 4.1.3, in order to analyze the effect of the DNA sequence input style on plant trait prediction. As shown in Figure 5, the results display the MSE of FE-WDNA with different input modes, where blue solid bars represent the MSE based on the SNP sequence mode, while diagonal striped bars represent the MSE based on the all-sequence mode.

For traits such as PH, Oil, Protein, FT, MT, and Yield, the model using the all-sequence mode outperformed the one using the SNP sequence mode in terms of the MSE. Especially in terms of Oil, Protein, FT and MT prediction, the models using the all-sequence mode significantly outperformed those using the SNP sequence mode. This is mostly because these traits are complex traits governed by multiple genes, and using whole-genome information enables the combined effects of these genes to be more effectively captured, improving the prediction accuracy. However, for HSW, the model based on the SNP sequence mode performed slightly better, possibly because HSW may be controlled by a few specific loci, allowing the SNP sequence mode to effectively capture the relevant features. Overall, the model using the all-sequence mode outperformed the one using the SNP sequence mode for most traits, but, for traits controlled by a few specific loci, such as the hundred-seed weight (HSW), the partial DNA sequence around SNP can still provide competitive performance in some cases.

#### 2.2.2. Different Feature Vector Constructions for Whole-Genome Nucleotide Sequencing

**Figure 6:**
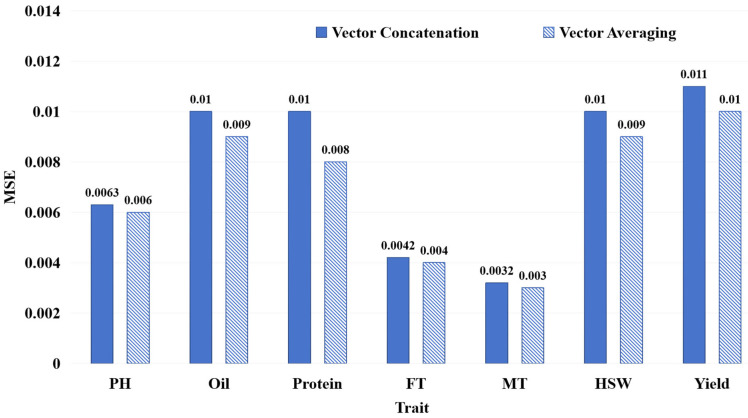
MSE of FE-WDNA with different modes of feature vector construction.

We conducted a comparative analysis to assess the impact of two feature fusion modes, as illustrated in Section 4.1.3, on FE-WDNA’s prediction performance. This is shown in Figure 6, where blue solid bars indicate the vector concatenation mode, and diagonal striped bars represent the vector averaging mode.

For all traits, the models using the vector averaging mode outperformed those using the vector concatenation mode. Based on these comparative results, we conducted exploratory analyses of the reason, considering the inter-chromosomal relationships and gene regulation. Previous research has indicated that Protein and Yield are complex, polygenic traits involving gene interactions across multiple chromosomes [20,21]. Traits such as FT and MT are closely related to the plant’s growth cycle, with gene loci distributed across different chromosomes, reflecting strong inter-chromosomal associations [22,23]. Oil synthesis and degradation involve multiple metabolic pathways and gene interactions [24,25]. Our analysis showed that the advantage of the vector averaging mode lies in its ability to reduce data dimensionality and lower model complexity while still capturing overall information, making it suitable for genome data with complex long-range dependencies and non-linear interactions in the genome.

#### 2.2.3. The Window Length of the Input Sequence and Feature Vector Dimension

**Figure 7:**
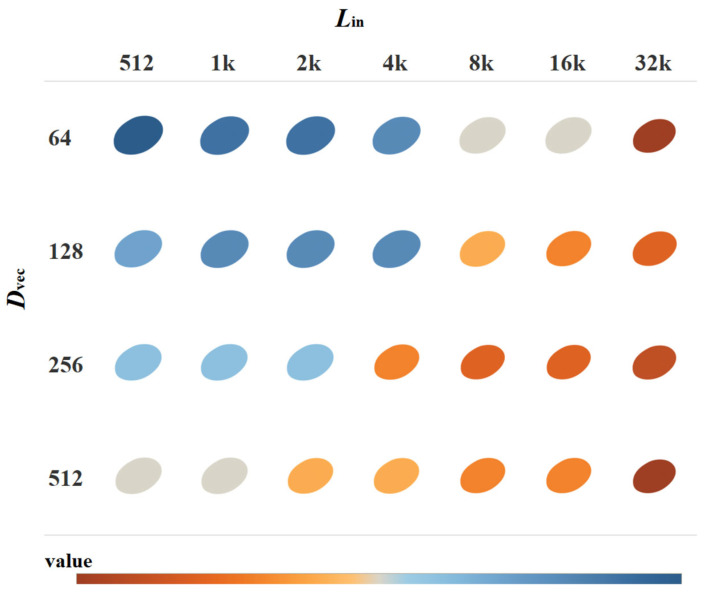
MSE of FE-WDNA with varying *L*_in_, and *D*_vec_.

Before the plant DNA sequences are processed through the FE-CDNA, they are divided by a context window length, referred to as *L*_in_. Then, after the sequence is put into FE-CDNA, it generates a feature vector, whose length is referred to as *D*_vec_. Both *L*_in_ and *D*_vec_ are crucial parameters influencing the performance of FE-WDNA and its trait prediction. This is shown in Figure 7.

Regarding the MT of soybeans, the results indicate that the model’s performance improves consistently as both *L*_in_, and *D*_vec_ increase. From the star-shaped heatmap distribution, it is clear that *L*_in_ has a significant impact on the model’s performance. As *L*_in_ increases incrementally from 512 to 32k, corresponding changes in performance are observed. For shorter *L*_in_ values (512 to 2k), the model demonstrates lower performance across different output dimensions, particularly in the 64- and 128-dimensional settings, where performance reaches the lowest (shaded in blue). This suggests that shorter *L*_in_ and lower *D*_vec_ values fail to capture sufficient genetic information, which limits the model’s predictive performance. As *L*_in_ extends to 4k and 8k, the model’s performance improves significantly, particularly in the 256- and 512-dimensional settings of *D*_vec_, where performance metrics rise markedly (as indicated by the transition from blue to orange). At the longest *L*_in_ (16k and 32k), the model achieves optimal performance across all dimensions of *D*_vec_ (shown by the deeper orange color). This indicates that longer input sequences allow the model to better capture long-range dependencies in the genome, which in turn enhances the predictive accuracy.

#### 2.2.4. Different Methods for Trait Prediction

**Figure 8:**
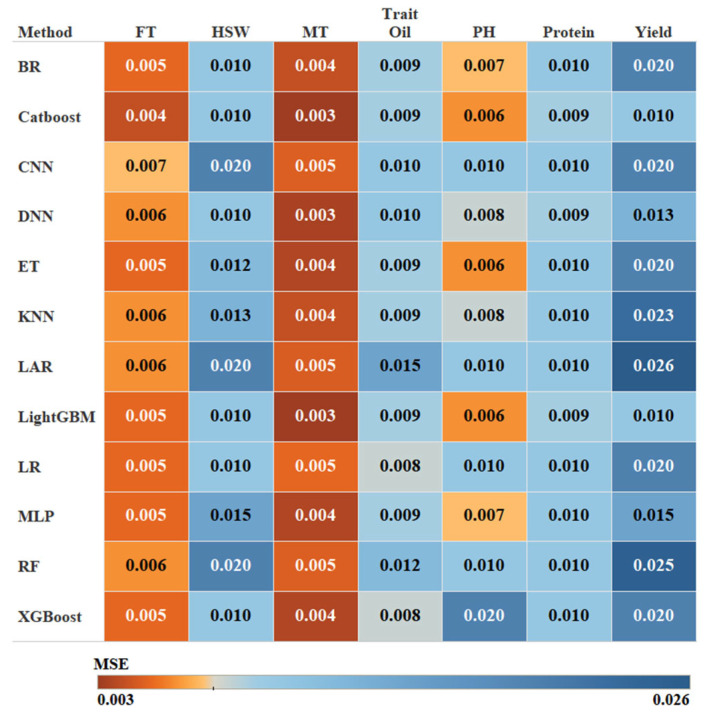
MSE of FE-WDNA using different trait prediction methods.

To evaluate the effectiveness of the proposed FE-WDNA model, we conducted parallel evaluations using nine conventional machine learning algorithms and three deep learning methods as trait prediction methods. These conventional machine learning models included Bayesian Ridge Regression (BR) [26], Catboost Classifier (Catboost) [27], Extra Trees (ET) [28], K-Nearest Neighbors (KNN) [29], Least Angle Regression (LAR) [30], Light Gradient Boosting Machine (LightGBM) [31], Logistic Regression (LR) [32], Random Forest Classifier (RF) [33], and Extreme Gradient Boosting (XGBoost) [34]. The deep learning methods included convolutional neural networks (CNN) [35], Multilayer Perceptron (MLP) [36], and deep neural networks (DNN) [37]. Each trait was trained using these models to facilitate a comparative analysis of their performance. The heatmap shown in Figure 8 displays the MSE of different methods based on FE-CDNA in predicting various soybean traits. The *X*-axis represents the phenotypic traits, and the *Y*-axis represents the trait prediction methods.

Gradient boosting models such as CatBoost and LightGBM performed exceptionally well across several traits, especially for FT and MT, where they achieved the lowest MSE values. These models are known for their ability to capture intricate feature interactions, making them ideal for phenotypic predictions based on DNA sequence data. ET also performed well, particularly for traits such as PH, where it achieved the lowest MSE due to its ability to manage high-dimensional and non-linear data. However, ET showed relatively weak performance for traits including Yield. Conversely, models such as RF struggled with nearly all traits. BR demonstrated average performance overall but exhibited relatively higher MSEs for Yield, reflecting its limitations in managing complex, non-linear datasets. The performance of the three deep learning algorithms fell short compared to traditional gradient-boosting algorithms. CNN delivers moderate results for the majority of traits, with relatively high prediction errors for complex traits such as Yield, Oil, and Protein. MLP and DNN exhibited similar abilities, performing slightly better for specific traits, such as MT and HSW, but the overall performance still did not surpass that of the gradient-boosting models. In future research, improvements in the performance of deep learning models may be achieved by increasing the dataset size and optimizing model architectures. The most optimal method was selected for each trait prediction with FE-WDNA, respectively.

#### 2.2.5. Sample Size for Trait Prediction Training

**Figure 9:**
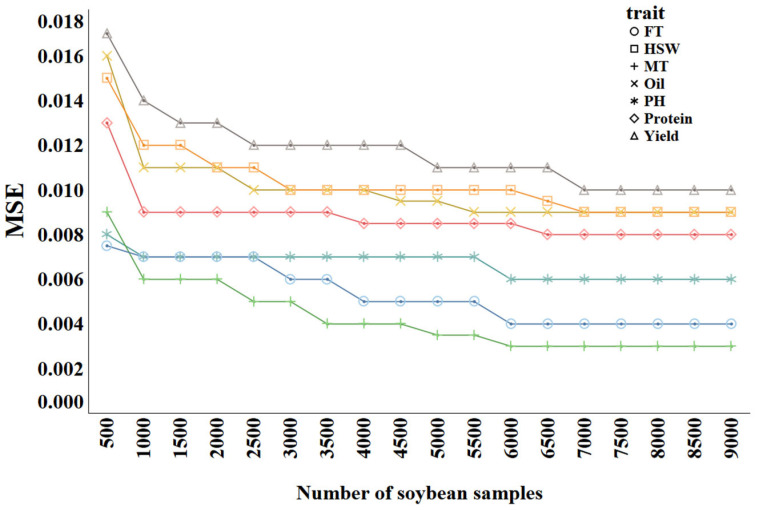
MSE of trait prediction methods with different training sample sizes.

The sample size directly affects the performance of prediction methods and is therefore a major factor to be considered in classic genomic selection approaches. To ascertain the optimal sample size for training the trait prediction model, we trained Catboost, which was generally the best model in Section 2.2.3; we used varying numbers of samples and monitored the predictive performance. The samples were divided into groups, ranging from 500 to 9000, in increments of 500. The results are shown in Figure 9, where different colors represent different traits.

As the sample size increases from 500 to 9000, the MSE values for all traits generally show a downward trend. Common patterns can be observed in the curves across different traits. In the early stage (500 to 2000 samples), the MSE decreases rapidly, indicating that increasing the sample size significantly improves the model’s performance. In the later stage (after 5000 samples), the rate of the MSE decrease slows, suggesting diminishing returns as the sample size grows. For example, Yield starts with a relatively high MSE of approximately 0.017. As the sample size increases to 2000, the MSE rapidly drops to around 0.013, and then continues to decrease to about 0.011 at 9000 samples. This demonstrates that increasing the sample size benefits Yield predictions, though the improvement diminishes in the later stages. Similarly, Protein begins with an MSE of about 0.013, which drops rapidly to approximately 0.009 in the first 2000 samples, stabilizing around 0.0085 as more samples are added. Traits such as Oil, PH, MT, and HSW exhibit similar trends. For FT, the MSE starts at 0.0075 and decreases to about 0.007 by 1000 samples, almost remaining stable thereafter, indicating that FT is relatively easier to predict, with the model achieving good performance with fewer samples. Overall, for complex traits such as Yield, Protein, and Oil, increasing the sample size is especially important; meanwhile, for simpler traits such as FT, fewer samples are required to achieve good performance.

## 3. Discussion

A key aspect of trait prediction lies in data feature engineering. We developed an LLM-based feature engineering technique, FE-WDNA, which is capable of identifying complex relationships embedded within DNA sequences. We explored the applications of FE-WDNA in predicting crop agronomic traits and compared its performance with other genomic prediction methods based on SNP data.

Notably, no existing methods utilize nucleotide resolution to enhance predictive accuracy. FE-WDNA effectively extracts DNA sequence features at nucleotide resolution. We tested several strategies to optimize the model and maximize its performance. The results demonstrated that FE-WDNA exhibits strong feature-extraction capabilities for complex quantitative traits such as Oil, Protein, and Yield of soybean. This strength likely stems from its model structure, which better captures whole-genome sequence representations and long-range dependencies. Currently, the context window of the input DNA sequence for training the FE-WDNA model is limited to 30k bp nucleotides, which is significantly shorter than the maximum sequence length (1 M) that the HyenaDNA framework can accommodate. Additionally, the feature vector dimension generated by FE-WDNA is only 512. These factors suggest significant potential for further performance improvements, which will be the focus of future research.

There is growing interest in LLM-based approaches for large-scale breeding programs to enhance genetic gains for crop traits. In this study, we introduced FE-WCDA, a novel method for constructing the feature engineering for DNA sequences. We evaluated its performance in soybean trait prediction against three widely used methods (SoyDNGP, DeepGS, and DNNGP). FE-WDNA demonstrated several advantages: (1) it efficiently handles DNA sequence data at nucleotide resolution, (2) it performed comparably with or better than commonly used models in plant trait prediction, and (3) the feature vector produced by the FE-WDNA feature engineering model can be easily analyzed on a local machine and integrated into various downstream models to suit specific tasks.

This research holds significant relevance in the current era of exponential growth in plant sequencing data and the relative scarcity of phenotype data. The application of LLMs to plant breeding is an emerging and active research area. The development and application of FE-WDNA in this study provide valuable insights for breeding-related research into the use of DNA sequence-based LLMs.

## 4. Methods

### 4.1. Design of FE-WDNA

#### 4.1.1. Algorithmic Frameworks

**Figure 10:**
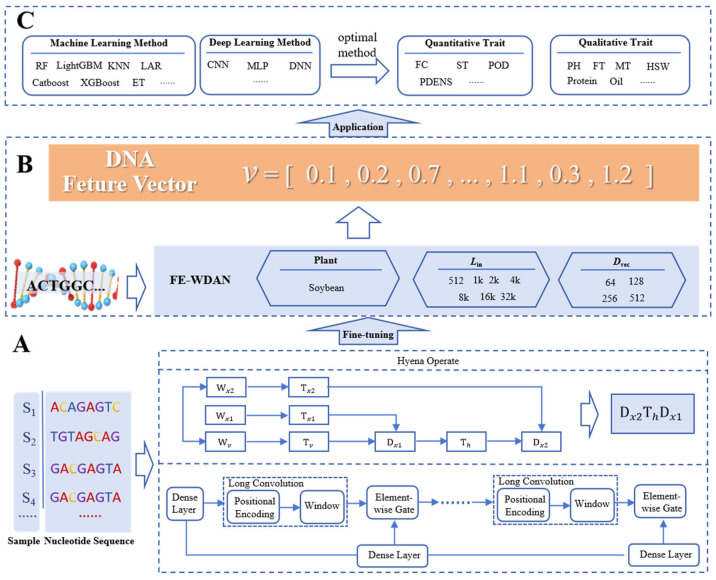
Illustration of the algorithmic frameworks used in FE-WDNA. (**A**) Different stages of FE-WDNA, including fine-tuned model construction; (**B**) feature vector generation; (**C**) plant agronomic trait prediction. FE-CDNA, a feature engineering model based on corn DNA sequences; CNN, convolutional neural network; MLP, multilayer perceptron; DNN, deep neural networks; RF, random forest; SVM, support vector machine; KNN, K nearest neighbor; FC, flower color; ST, stem termination; POD, pod color; PDENS, pubescence density; PH, plant height; FT, flowering time; MT, maturity time; HSW, hundred-seed weight.

Figure 10 illustrates the framework of the FE-WDNA method, which is divided into three components: A, B, and C. Component A describes the process of constructing FE-WDNA based on HyenaDNA. As depicted in Figure 10A, the FE-CDNA model utilizes soybean DNA sequence data to fine-tune the improved HyenaDNA model. HyenaDNA is a decoder-only, sequence-to-sequence architecture defined by a stack of blocks built upon the Hyena operator. The pre-training objective of HyenaDNA is to predict the next nucleotide, which facilitates the elucidation of complex relationships between nucleotides. To achieve this, we adopt the natural DNA vocabulary and tokenize the DNA sequence, representing each nucleotide as a token. The tokens include “A”, “T”, “C”, and “G”, along with special tokens for padding, separation, and unknown characters. These tokens serve as the input for the fine-tuning process of HyenaDNA. The window length of the DNA input sequence (*L*_in_) and the dimension of the output feature vector (*D*_vec_) are key hyperparameters for training FE-WDNA. We experimented with *L*_in_ values of 512, 1k, 2k, 4k, 8k, 16k, and 32k, and *D*_vec_ values of 64, 128, 256, and 512.

Component B involves inputting whole-genome DNA sequences into FE-WDNA to obtain their corresponding feature vectors. Once the DNA language model is trained, it is applied to plant DNA sequences to extract high-dimensional feature vectors. These vectors represent a space where genomic relationships become more discernible, providing a foundation for downstream applications.

Component C focuses on the application of FE-WDNA. Using the feature vectors derived from FE-WDNA, a supervised learning approach is employed to predict plant traits by integrating machine learning and deep learning methods. The predictive model is trained on a dataset comprising DNA feature vectors paired with corresponding phenotypic outcomes, enabling the model to learn the mapping from genomic data to phenotypic traits. In this study, several predictive models, including random forest (RF), LightGBM, CatBoost, and convolutional neural networks (CNNs), were trained to evaluate the performance of FE-WDNA. For each trait, the most optimal model was selected to provide the final predictive results.

#### 4.1.2. HyenaDNA

HyenaDNA builds on the principles of Hyena. Hyena is an implicit convolutional language model; it has been shown to match the performance of attention mechanisms in terms of quality, while significantly reducing computational complexity [38]. This reduction enables the more efficient processing of longer context windows. Hyena leverages a parameter-efficient global convolutional filter combined with a data-driven gating mechanism, allowing for context-specific operations on individual tokens. Historically, convolutions have played a pivotal role in deep learning. Recent research has demonstrated that stacking multiple long convolutional layers can achieve state-of-the-art performance on various benchmarks involving long sequences, such as those found in the long-range arena (LRA) [39,40,41]. As previously discussed, Hyena’s core operations unlock the potential to capture both long-range dependencies and single-nucleotide resolution in real genomic sequences.

Each Hyena operator comprises long convolutions and element-wise gating layers. The gating mechanism receives projections of the input through dense layers and short convolutions, while the long convolutions are implicitly parameterized by a multilayer perceptron that generates the convolutional filters. The convolution itself is computed via the Fast Fourier Transform. A Hyena operator can be defined as shown in Figure 10A. In this figure, *x*_1_, *x*_2_, and *v* represent the input projections, while $T_{h}\in R^{L\times L}$ is the Toeplitz matrix constructed from the long convolution filter generated by a neural network. The filter values are derived from a small neural network that takes the position index and optional positional encoding as the input, enabling the operator to process extremely long sequences without a linear increase in the number of parameters.

The architecture of HyenaDNA is built upon a straightforward stack of Hyena operators and has demonstrated outstanding performance across a variety of tasks. Drawing inspiration from HyenaDNA, its nucleotide-level resolution introduces novel opportunities for genomic research. However, the current implementation of HyenaDNA is primarily tailored for processing a single human whole-genome sequence. It generates feature vectors only for partial DNA sequences, lacking a comprehensive framework for whole-genome DNA sequence analysis. To overcome this limitation, we enhanced and refined the HyenaDNA method, developing a feature engineering framework, which is called the FE-WDNA and was specifically designed for plant whole-genome DNA sequences, with soybean as a representative case study. The FE-WDNA was trained on 8 NVIDIA A100 GPUs, and these GPUs were sourced from the Beijing branch of NVIDIA Corporation, which is located in Beijing, China. The main hyperparameters are presented in Table 1.

#### 4.1.3. Whole-Genome Feature Vector Construction

The representation of DNA sequences is the most critical factor influencing the performance of FE-WDNA. Two key considerations in achieving the representation of high-quality DNA sequences are how to transform the nucleotide sequence for the model and how to construct the whole-genome feature vector for a single plant sample. To address these, we systematically explore various modes for both factors.

(1) Different DNA sequence input modes

**Figure 11:**
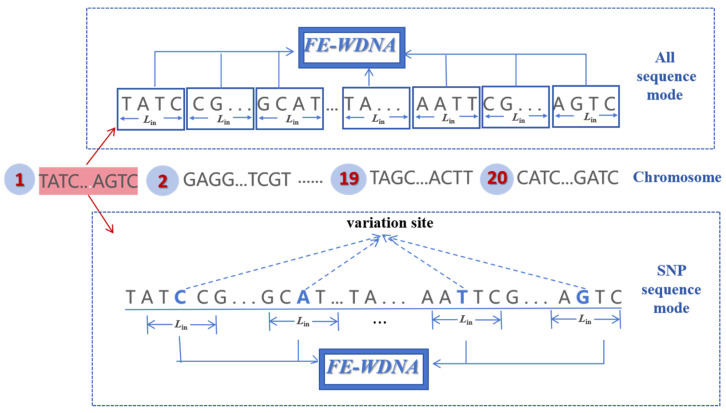
Overview of different DNA sequence input modes for FE-WDNA.

Prior to inputting DNA data into the model, the DNA sequences must be segmented based on the defined window length, which is defined as *L*_in_. We explored two types of DNA sequence inputs in FE-WDNA: the first including all nucleotides in the whole-genome DNA sequences (referred to as the all-sequence mode), and the second comprising partial DNA sequence extracted from the nucleotides around the variate sites in the SNPs (referred to as the SNP sequence mode), as shown in Figure 11. For the all-sequence method, the entire genomic DNA sequence is segmented into fragments of length *L*_in_, and each segment is input into the FE-WDNA model. For the SNP sequence mode, nucleotide sequences of length *L*_in_ are extracted around each variation site in the SNP data, with the *L*_in_/2 nucleotide sequence on either side of the variation site.

(2) Different feature constructions for all chromosomes in the whole-genome nucleotide sequence

**Figure 12:**
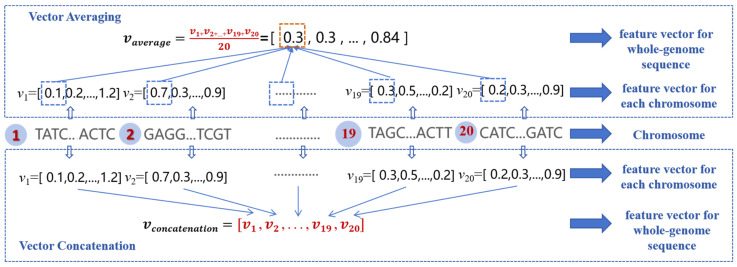
Feature vector construction for whole DNA sequences of a crop sample.

A plant sample of soybean includes 20 chromosomes, and FE-WDNA generates one feature vector for every chromosome. To construct a comprehensive feature vector for a plant sample, we propose two feature fusion methods, as illustrated in Figure 12. The first method is the equal-weighted averaging fusion (referred to as vector averaging), where 20 feature vectors are summed and averaged. The second method is the concatenation fusion (referred to as vector concatenation), in which the feature vectors from all chromosomes are concatenated to form a longer feature vector.

### 4.2. Datasets Used for Genomic Prediction

The data used for training and prediction with the FE-WDNA model were sourced from two comprehensive online datasets related to the genotypic and phenomic data of soybeans. The genotypic information was obtained for an extensive collection of 20,087 soybean accessions from the SoyBase dataset, which is from the USDA Soybean Germplasm Collection and includes 42,509 high-confidence SNPs based on the SoySNP50K iSelect BeadChip. Phenotypic data for these selected soybean accessions were retrieved from the GRIN Global database (https://npgsweb.ars-grin.gov/gringlobal/search, accessed on 10 May 2024). From the original 23 agronomic traits, we selected 11 key traits for analysis, including seven quantitative traits (protein content, oil content, hundred-seed weight, flowering date, maturity date, yield, and plant height) and four qualitative traits (stem termination, flower color, pubescence density, and pod color).

**Figure 13:**
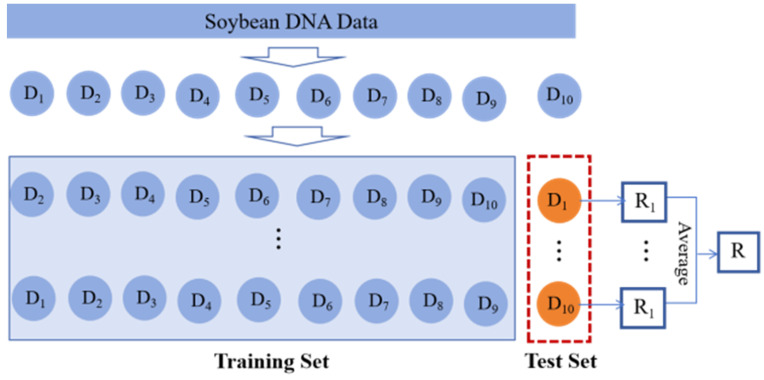
Ten-fold cross-validation approach.

We selected 1000 samples as the fine-tuning data for HyenaDNA and randomly chose 6950 samples from the remaining dataset for the phenotypic trait prediction. For each qualitative trait, samples with missing trait values and categories with insufficient sample sizes were removed. The model was then trained using a 10-fold cross-validation approach, as illustrated in Figure 13. For each quantitative trait, samples with missing trait values were removed, and the model was trained using the same 10-fold cross-validation approach, following a process similar to that used for the qualitative traits.

The cross-validation process begins by randomly partitioning the dataset into 10 independent subsets of approximately equal size. Each subset was sequentially selected as the test set, while the remaining nine subsets served as the training set, forming 10 distinct training–testing splits. This process resulted in 10 rounds of model training and evaluation. The final performance metric for the model was calculated as the average result from these 10 rounds of testing.

For the qualitative traits, the number of valid samples for each trait after filtering is summarized in Table 2. Traits with categories removed due to insufficient sample sizes are highlighted with a red background in the table.

**Figure 14:**
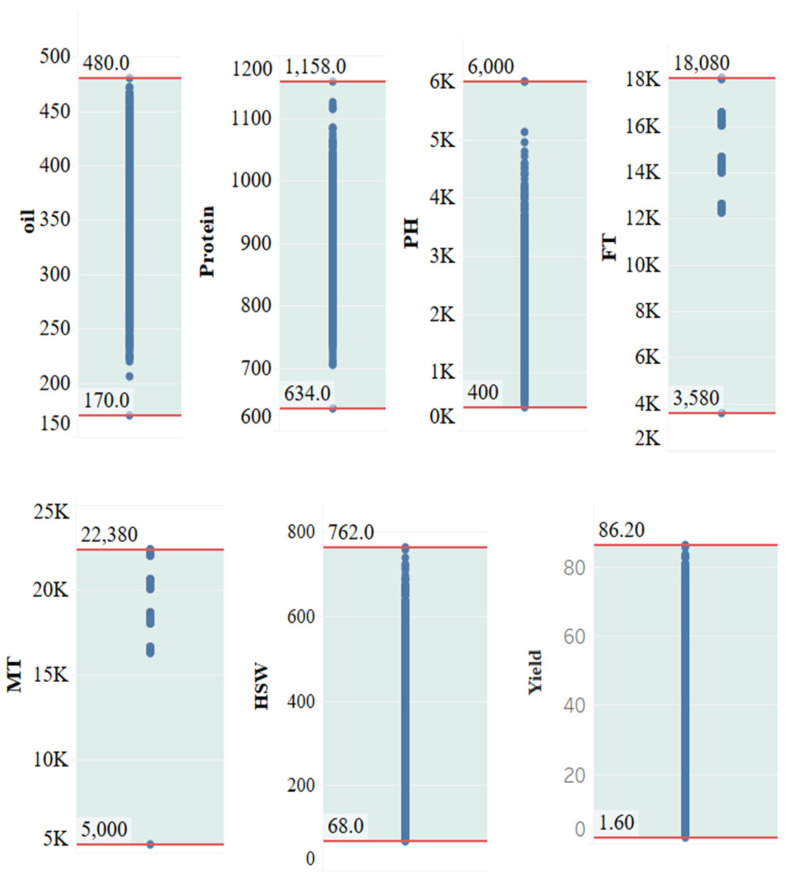
Value range distribution of quantitative traits. PH, plant height; FT, flowering time; MT, maturity time; HSW, hundred-seed weight; MSE, mean standard error.

For the quantitative traits, the distribution of trait values is shown in Figure 14, where the maximum and minimum values are indicated by red horizontal lines.

### 4.3. Methods Used for the Comparison with FE-WDNA

To evaluate the performance of FE-WDNA, we used soybean trait prediction as a case study and conducted comparative experiments with three state-of-the-art (SOTA) methods in plant genetic prediction: SoyDNGP [42], DeepGS [43], and DNNGP [44]. The evaluation included both qualitative and quantitative trait predictions. Moreover, we investigated the impact of different DNA sequence input formats, various feature construction approaches across all chromosomes within the whole genome, and additional factors that might influence FE-WDNA’s performance. The baseline models were implemented using Python 3.8 following the detailed descriptions given in their respective original research studies. To ensure fairness, all models, including FE-WDNA, were trained and tested on the same dataset.

(1) DNNGP

DNNGP is a deep learning approach that utilizes a convolutional neural network (CNN) framework, with SNP data as the input. Its architecture comprises an input layer, three convolutional layers, a batch normalization layer, two dropout layers, a flattening layer, a dense layer, and an output layer. The input layer processes data as an *n* × *m* matrix, where *n* represents the number of individuals and *m* denotes the number of markers. The majority of computations are performed in the three convolutional layers, which serve as the network’s core.

(2) DeepGS

DeepGS adopts a CNN-based structure for genetic prediction. Its architecture includes an input layer, a CNN layer, a sampling layer, three dropout layers, two fully connected layers, and an output layer. The rectified linear unit (ReLU) activation function is employed in both the CNN layer and the first fully connected layer, facilitating effective feature extraction and learning.

(3) SoyDNGP

SoyDNGP is inspired by the segmentation framework of the VGG deep learning network [45]. Its design revolves around convolutional blocks, each consisting of a convolutional layer, a normalization layer, and a ReLU activation layer. Feature extraction units in the network are constructed by combining one or two convolutional layers, followed by fully connected layers. To mitigate overfitting in deeper architectures, dropout layers are introduced after each convolutional block. The complete network includes 12 convolutional layers and a single fully connected layer.

### 4.4. Assessment Metrics

(1) Quantitative trait prediction

We used the mean squared error (MSE), the mean absolute error (MAE), and Pearson’s correlation coefficient (r) as evaluation metrics for quantitative trait prediction. To calculate the MSE and MAE values, we trained the trait prediction methods using the normalized trait values as inputs. For the calculation of the PCC values, we trained the trait prediction methods using the original trait values as inputs.

(1) $$MSE=\frac{1}{N}\sum_{n=1}^{N}{\left(y_{n}-\hat{y_{n}}\right)}^{2}$$

The MSE is a widely used metric for measuring the average squared difference between the predicted and actual trait values for all crop samples, where lower values indicate better predictive performance. It provides an indication of the model’s prediction accuracy, where a lower MSE corresponds to higher accuracy. *N* refers to the total number of crop samples in the dataset, *y_n_* denotes the predicted trait value for the *n*th crop sample, and $\hat{y_{n}}$ represents the observed trait value for the *n*th crop sample.

(2) $$MAE=\frac{1}{N}\sum_{n=1}^{N}\left|y_{n}-\hat{y_{n}}\right|$$

The mean absolute error (MAE) is a commonly used metric for evaluating the average magnitude of errors between predicted and actual trait values across all crop samples. Unlike the mean squared error (MSE), the MAE measures the absolute differences without emphasizing larger errors, making it less sensitive to outliers. Lower MAE values indicate better predictive performance. The MAE provides a straightforward and interpretable measure of prediction error, with a lower value corresponding to a more accurate model. *N* refers to the total number of crop samples in the dataset, *y_n_* denotes the predicted trait value for the *n*th crop sample, and $\hat{y_{n}}$ represents the observed trait value for the *n*th crop sample.

(3) $$r=\frac{\sum_{n=1}^{N}\left(y_{n}-\bar{y})(\hat{y_{n}}-\bar{\hat{y}}\right)}{\sqrt{\sum_{n=1}^{N}{\left(y_{n}-\bar{y}\right)}^{2}}\sqrt{\sum_{n=1}^{N}{\left(\hat{y_{n}}-\bar{\hat{y}}\right)}^{2}}}$$

(4) $$\bar{y}=\frac{1}{N}\sum_{n=1}^{N}y_{n}$$

(5) $$\bar{\hat{y}}=\frac{1}{N}\sum_{n=1}^{N}\hat{y_{n}}$$

The Pearson’s correlation coefficient (PCC) quantifies the strength and direction of the linear relationship between the predicted and actual crop trait values. It ranges from −1 to 1, where a higher *r* corresponds to higher accuracy. This metric is commonly used to assess the predictive accuracy of models in trait prediction studies. *N* refers to the total number of crop samples, *y_n_* and $\hat{y_{n}}$ denote the observed and predicted crop trait values for the *n*th sample, respectively, and $\bar{y}$ and $\bar{\hat{y}}$ represent the means of the observed and predicted crop trait values, respectively.

(2) Qualitative trait prediction

(6) $$Accuracy=\frac{TP+TN}{TP+TN+FP+FN}$$

We used prediction accuracy for qualitative trait classification tasks. Accuracy represents the proportion of correctly classified samples (both positive and negative) out of the total number of crop samples. It is one of the most commonly used evaluation metrics in classification tasks. TP (true positive) denotes the number of positive samples correctly classified as positive, TN (true negative) represents the number of negative samples correctly classified as negative, FP (false positive) denotes the number of negative samples incorrectly classified as positive, and FN (false negative) represents the number of positive samples incorrectly classified as negative.

## 5. Conclusions

Previous studies typically relied on SNP data for plant phenotype analysis. While SNPs represent important genetic variation extracted from DNA sequences, they are often incomplete and provide insufficient genetic information. This limitation motivated our exploration of whole-genome DNA sequence-based genomic prediction using LLMs.

We introduced FE-WDNA, a novel feature engineering method for plant DNA sequencing at single-nucleotide resolution. This model is capable of learning generalizable features that can be fine-tuned for various tasks, such as trait prediction and the identification of regulatory elements. The performance of FE-WDNA in soybean trait prediction was evaluated against three widely used prediction methods—SoyDNGP, DeepGS, and DNNGP. The results demonstrate that FE-WDNA is a promising and practical approach that effectively incorporates DNA sequence data for the prediction of agronomic traits. Our study aims to shed light on how LLM can be applied in plant whole-genome feature construction and contribute to improving genomic selection strategies.

Phenotypic trait prediction serves only as one downstream task in demonstrating the performance of FE-WDNA. The feature vectors generated by FE-WDNA can support a variety of downstream tasks by serving as inputs for different predictive models. For example, in DNA structure analysis, these tasks include the prediction of core promoters and open chromatin, while, in DNA functionality, they include the prediction of sequence conservation and histone modifications.

In future research, we aim to optimize FE-WDNA in two key respects: (1) enhancing the model’s capacity to engineer features from DNA sequences by increasing the context window of the input DNA sequence and the dimensionality of the feature vectors; and (2) expanding its applications to unlock the model’s full potential in additional downstream tasks.

## Figures and Tables

**Table 1:** Hyperparameters in the process of fine-tuning FE-WDNA.

Hyperparameter	Value
batch_size	16
learning_rate	6 × 10^−4^
max_epochs	6
n_layer	2
dimension_model of output	256
max_length of input	32,768

**Table 2:** Category distribution of qualitative traits.

Trait		Number
FC	P	3227
	W	1631
	Dp	30
	B	5
	Lp	5
	Pth	4
ST	D	2644
	N	1851
	S	407
P_DENS	N	2938
	Ssp	1902
	Sp	37
	G	12
	Sdn	7
	Dn	6
POD	Br	3242
	Tn	1287
	BI	235
	Dbr	109
	Lbr	28
	H	1

The orange background indicates the data that was removed from the dataset due to the small sample size.

## Data Availability

The original data presented in the study are openly available in the GRIN Global database at https://npgsweb.ars-grin.gov/gringlobal/search (accessed on 10 May 2024).
